# What drives us apart? Decomposing intersectional inequalities in cigarette smoking by education and sexual orientation among U.S. adults

**DOI:** 10.1186/s12939-019-1015-1

**Published:** 2019-07-17

**Authors:** Nada Amroussia, Jennifer L. Pearson, Per E. Gustafsson

**Affiliations:** 1 Division of Social and Behavioral Health, University of Nevada, Reno, USA; 2 Division of Social and Behavioral Health/Health Administration and Policy, University of Nevada, Reno, USA; 3 Department of Health, Behavior, and Society, Johns Hopkins Bloomberg School of Public Health, Baltimore, MD USA; 4 Department of Public Health and Clinical Medicine, Umeå University, Umeå, Sweden

**Keywords:** Sexual and Gender minorities, Education, Cigarette smoking, Intersectionality, Health inequality, Blinder-Oaxaca decomposition

## Abstract

**Background:**

Socio-economic and sexual orientation inequalities in cigarette smoking are well-documented; however, there is a lack of research examining the social processes driving these complex inequalities. Using an intersectional framework, the current study examines key processes contributing to inequalities in smoking between four intersectional groups by education and sexual orientation.

**Methods:**

The sample (28,362 adults) was obtained from Wave 2 (2014–2015) of the Population Assessment of Tobacco and Health (PATH) Study. Four intersectional positions were created by education (high- and low-education) and sexual orientation (heterosexual or lesbian, gay, bisexual, or queer/questioning (LGBQ). The joint inequality, the referent socio-economic inequality, and the referent sexual orientation inequality in smoking were decomposed by demographic, material, tobacco marketing-related, and psychosocial factors using non-linear Oaxaca decomposition.

**Results:**

Material conditions made the largest contribution to the joint inequality (9.8 percentage points (p.p.), 140.9%), referent socio-economic inequality (10.01 p.p., 128.4%), and referent sexual orientation inequality (4.91 p.p., 59.8%), driven by annual household income. Psychosocial factors made the second largest contributions to the joint inequality (2.12 p.p., 30.3%), referent socio-economic inequality (2.23 p.p., 28.9%), and referent sexual orientation inequality (1.68 p.p., 20.5%). Referent sexual orientation inequality was also explained by marital status (20.3%) and targeted tobacco marketing (11.3%).

**Conclusion:**

The study highlights the pervasive role of material conditions in inequalities in cigarette smoking across multiple dimensions of advantage and disadvantage. This points to the importance of addressing material disadvantage to reduce combined socioeconomic and sexual orientation inequalities in cigarette smoking.

## Background

Socio-economic as well as sexual orientation inequalities in cigarette smoking are well-documented among U.S. adults [1–9]. Cigarette smoking prevalence is significantly higher among people with low socio-economic status (SES) [1–5]; in 2018, 23.1% of adults without a high school degree were current smokers, compared to 10.7% of adults who had an undergraduate degree [5]. Similarly, lesbian, gay, bisexual, and queer/questioning (LGBQ) adults are disproportionately burdened by cigarette smoking, with 20.3% LGBQ adults reporting current smoking in 2018 compared to only 13.7% of heterosexual adults [1, 5–8]. These inequalities in smoking could potentially be reflected in inequalities in smoking-attributed morbidity and mortality disadvantaging low SES and LGBQ adults [9].

Socio-economic inequalities and sexual orientation inequalities in cigarette smoking have conventionally been understood, studied, and addressed as separate axes of inequalities [10–17]. Such a singular and fragmented approach does not capture how these two axes of inequalities interplay to affect adults’ smoking status, as challenged by the framework of *intersectionality*. The intersectional framework is increasingly used in public health research to elucidate the complexity of health inequalities [18–20]. Intersectionality assumes that people’s social positions are shaped by interlocking rather than separate axes of power relations stemming from, mutually constructed social factors, including (among others,), sexual orientation, socio-economic status, race, and gender. These interlocking power relations create a complex web of social inequalities determining people’s advantage and disadvantage [18–20]. According to the intersectional approach, an individual’s experience and his/her health, “are not simply the sum of their parts” [21]; for example, the health and the implications of being an LGBQ adult differ between low-educated LGBQ adults and high-educated LGBQ adults [21].

Intersectionality considers that social inequalities can be reinforced or contested through different social processes of oppression or privilege [21]. In this sense, the intersectional framework allows examining health inequalities not only at the intersection of multiple social positions (e.g. the intersection of sexual orientation and socio-economic status), but also at the intersections of different social processes (e.g. material disadvantage and sexual orientation-based discrimination). This has the potential to yield a deeper, more specific, and realistic understanding of health inequalities [21]. To the authors’ knowledge, however, only a few studies have examined intersectional inequalities in smoking [22–24]. These studies have pointed out the important role of the intersections of sexual orientation, with gender, age, gender identity, and to a lesser extent race/ethnicity in explaining the patterns of cigarette smoking among U.S. adults and youth [22–24]. In a recent study (Amroussia N, Gustafsson PE, Pearson JL: Do inequalities add up? Intersectional inequalities in smoking by sexual orientation and education among U.S. adults, unpublished), we found complex patterns of cigarette smoking among U.S. adults at the intersection of sexual orientation and education, thereby, socio-economic and sexual orientation inequalities in cigarette smoking do not add up in expected patterns. This small collection of studies illustrate the unique and policy-relevant knowledge gained from considering axes of inequality as complex rather than disentangled phenomena.

A key challenge is understanding the underlying social processes that may generate, amplify or temper inequalities between complex social positions [21], as this evidence would enable generating evidence necessary to develop tailored and effective smoking prevention programs and policies. However, attempts to explain the combination of socio-economic and sexual orientation inequalities in cigarette smoking are rare [15, 17]. Drawing on the literature on socioeconomic inequalities and sexual orientation inequalities in smoking, access to material and social resources (e.g. high income and access to social support) might counteract socio-economic and sexual orientation inequalities in cigarette smoking [10, 11, 13, 14, 17, 25], but other oppressive processes (e.g. financial stress, lack of health insurance, and tobacco marketing strategies targeting disadvantaged sub-populations), might exacerbate these inequalities [10, 26–30]. However, how these social processes may play out in the context of interacting and complex inequalities across socio-economic status and sexual orientation have not been studied previously.

Building on our recent study that examined inequalities in cigarette smoking at the intersection of sexual orientation and education (Amroussia N, Gustafsson PE, Pearson JL: Do inequalities add up? Intersectional inequalities in smoking by sexual orientation and education among U.S. adults, unpublished), the aim of the current study is to examine key processes contributing to these inequalities, using non-linear Blinder-Oaxaca decomposition method. Blinder-Oaxaca decomposition analysis method has gained attention in recent years in health inequalities’ research, including research on intersectional inequalities [31], as it allows not only quantifying inequalities in health between two distinct groups, but also attributing this inequality to the unequal distribution of individual factors [32].

## Methods

### Study population

The sample was drawn from the Wave 2 Population Assessment of Tobacco and Health (PATH) Study, conducted between October 2014 and October 2015. The PATH Study is a nationally representative longitudinal cohort study of non-institutionalized US adults and youth aged 12 years and older [33]. The initial sample included 45,971 US adults and youth, and the Wave 2 sample consisted of 28,362 adults ages 18 and over. The weighted retention rate of Wave 2 adult interviews was 83.1% [34].

A four-stage, stratified probability sample design was employed with oversampling young adults (18–24 years), African Americans, and adult tobacco users. Information on tobacco use behavior, attitudes and beliefs, as well as tobacco-related health outcomes were collected using Audio-Computer Assisted Self-Interviews [33].

### Measures

#### Outcome: current cigarette smoking

Current cigarette smoking was operationalized as “yes” if the participants fulfilled both of the following two conditions: 1) reported current cigarette use on “every day” or “some days”; as well as 2) smoked more than 100 cigarettes in their lifetime [34].

#### Exposure: intersectional positions by sexual orientation and socio-economic status

Adult sexual orientation was based on the item “Do you consider yourself to be (1) straight, (2) lesbian or gay, (3) bisexual, or (4) something else?” A dichotomous variable (LGBQ adults vs. heterosexual adults) was created by grouping the three categories “lesbian or gay”, “bisexual”, and “something else” into one category. Education was used as an indicator of SES and was categorized into two categories: “less than high school diploma” vs. “high school diploma or more”. The cut-off point of high school diploma was chosen as previous research has shown that adults with less than high school education are disproportionally burdened by cigarette smoking [5]. The terms “low educated” and “high educated” will be used to refer to “less than high school diploma” vs. “high school diploma or more” respectively.

Based on sexual orientation and education, four mutually exclusive intersectional positions were formed: high educated heterosexual adults defined as the doubly advantaged group; low-educated heterosexual adults; high-educated LGBQ adults; and low-educated LGBQ adults, defined as the doubly disadvantaged group.

In our previous paper (Amroussia N, Gustafsson PE, Pearson JL: Do inequalities add up? Intersectional inequalities in smoking by sexual orientation and education among U.S. adults, unpublished) and following Jackson et al. method [35], three intersectional inequalities were defined: the *joint inequality* was defined as the inequality in current cigarette smoking between the doubly disadvantaged group (low-educated LGBQ adults) and the doubly advantaged group (high-educated heterosexual adults); the *referent socio-economic inequality* as the inequality in current cigarette smoking between low-educated heterosexual adults and high-educated heterosexual adults; and the *referent sexual orientation inequality* as the inequality in current cigarette smoking between high-educated LGBQ adults and high-educated heterosexual adults. The results of our previous study indicated that these inequalities were positive and of substantial size, suggesting the importance of examining factors and processes contributing to these inequalities.

#### Explanatory factors: processes of privilege and oppression

Following the intersectional framework [21], processes of oppression and privilege that might reinforce or mitigate health inequalities were identified. Three factor-groups were chosen to assess these processes. These groups reflected *material conditions*, *tobacco marketing-related factors*, and *psychosocial factors*.

Material conditions were measured using five variables: annual household income, employment status, receiving assistance, housing, and health insurance. Participants were asked “which of the following categories best describes your total household income in the past 12 months?”, and a derived variable was created by dividing income into five groups: <$10,000, $10,000–$24,999, $25,000–$49,999, $50,000–$99,999, and $100,000 or more, according to the PATH study codebook [36]. Employment status was categorized into “full-time”, “part-time”, and “unpaid”, with full-time employment considered the most advantaged groups as it is more likely to provide benefits, such as health insurance, and also higher income. Receiving assistance was coded as “yes” and “no”. Housing was based on the item “do you own or rent your home?” and was categorized into “owned”, “rented”, and “something else”, reasoning that those who owns their dwelling have a financial security compared to those who rent. Health insurance was categorized into “private insurance”, “Medicaid/Medicare and other insurance”, and “no insurance”, with adults having private insurance representing the most advantaged group, as private insurance in the U.S. is both more costly and tend to involve greater coverage and benefits.

Tobacco marketing-related factors were measured using three variables chosen to capture and differentiate the current major forms of tobacco marketing in the U.S.: exposure to contextually-targeted tobacco advertisements in the physical environment, exposure to individually-targeted tobacco marketing through direct mailings of coupons or promotions, and exposure to tobacco advertisements in media such as newspapers and websites.

Exposure to contextual-level targeting advertisement (yes vs. no) was based on two items: “in past 30 days, noticed cigarettes or other tobacco products being advertised: at events such as fairs, festivals, or sporting events” and “in past 30 days, noticed cigarettes or other tobacco products being advertised: on posters or billboards”. Exposure to individual-targeted tobacco marketing (yes vs. no) was assessed through the item “In past 12 months, received promotions or coupons in the mail for cigarettes or tobacco products”. Exposure to advertisement on media (yes vs. no) was based on four items: “in past 30 days, noticed cigarettes or other tobacco products being advertised: in newspapers or magazines”, “in past 30 days, noticed cigarettes or other tobacco products being advertised: on websites or social media sites”, “in past 30 days, noticed cigarettes or other tobacco products being advertised: on radio”, and “in past 30 days, noticed cigarettes or other tobacco products being advertised: on television”.

Psychosocial factors were measured perceived quality of life and perceived satisfaction with social relationships and activities, both of which were categorized in a comparable manner into good, moderate, and poor quality of life and satisfaction, respectively. Participants were asked “in general, would you say your quality of life” with five response options “excellent”, “very good”, “good”, “fair”, and “poor”. The categories “excellent”, “very good”, “good” were collapsed into one category to create a large reference group for the three-level variable perceived quality of life: “poor,” “fair,” and “good”, with adults reporting poor perceived quality of life representing the most disadvantaged group. Perceived satisfaction with social relationships and activities was based on the item “in general, how satisfied are you with your social activities and relationships?” with five response options “extremely satisfied”, “very satisfied”, “moderately satisfied”, “a little satisfied”, and “not all satisfied”. Similar to the quality of life variable, the categories “extremely satisfied”, “very satisfied” were collapsed into one category “very satisfied” to generate a large reference group, and the categories “moderately satisfied” and “a little satisfied” were collapsed into one category “moderately satisfied”, resulting in the final three-level variable perceived satisfaction with social relationships and activities (“very satisfied,” “moderately satisfied,” and “not at all”, with adults reporting feeling unsatisfied with social relationships are the most disadvantaged group).

#### Socio-demographic factors

Previous research suggests that both socio-economic inequalities and sexual orientation inequalities in cigarette smoking may vary by gender [37, 38], age [39], and race/ethnicity [40]. Additionally, marital status has been linked to cigarette smoking [41, 42]. Variables capturing these factors were, therefore, included in the analysis: gender (men vs. women), age (18–24, 25–44, and 45+), race/ethnicity (White non-Hispanic, Hispanic, and Non-White non-Hispanic), and marital status (married, separated/widow/divorced, and never married).

### Statistical analyses

The analysis consisted of three steps: two preliminary set of analyses and one set of main analysis. The first set consisted of descriptive analyses that comprised the distribution of sociodemographic factors and indicators of material, marketing and psychosocial processes across the four intersectional social positions, to descriptively illustrate any intersectional inequalities in social processes. The second set of analyses consisted of three multiple logistic regressions where current cigarette smoking was regressed on all social process variables as well as one of the intersectional inequality variables in each regression; the joint inequality, the referent socioeconomic inequality, or the sexual orientation inequality variable. A multicollinearity analysis was conducted to estimate the variance inflation factors (VIFs) for all the variables included in the models [43], using the command *vif* in STATA 15 (StataCorp, 2017). The results indicated that the variables included in the models were not highly collinear, with a max VIF of 1.65, mean VIF of 1.30, and highest VIF displayed by annual household income, marital status, and age (1.65, 1.55, and 1.54 respectively).

The proportion differences between intersectional groups illustrated in the first set of analyses, and the logit estimates from the second set of analyses are both used in the third set of analyses, which served to explain the joint inequality, the referent socio-economic inequality, and the referent sexual orientation inequality through non-linear Blinder-Oaxaca decomposition analysis [32, 44].

The non-linear Blinder-Oaxaca method is an extension of the original linear Blinder-Oaxaca decomposition [45, 46] and aims to explain the gap in a binary outcome variable (i.e. current cigarette smoking) between two groups (e.g. low-educated LGBQ adults and high-educated heterosexual adults for the joint inequality) using a set of explanatory variables [32, 44, 47]. The gap in the binary outcome variable (i.e. current cigarette smoking) is, then, decomposed into: i) an “explained” part attributable to the differences in the frequency of observed explanatory factors between the comparison groups, and ii) an “unexplained” part attributable to differences in the estimated coefficients [32, 45] or to differences in unobserved explanatory factors [44].

Non-linear decomposition analyses were performed separately for the joint inequality, referent socio-economic inequality, and referent sexual orientation. The set of explanatory factors included in the models consisted of all social processes’ indicators: material conditions, tobacco marketing-related factors, psychosocial factors, as well as socio-demographic factors. The absolute contribution and relative contribution of each factor to the inequalities were, then, computed. The relative contribution of each factor was estimated by dividing the absolute contribution of the factor (on the same scale as the inequality; prevalence difference) by the total explained component of the inequality.

The *oaxaca* command on STATA 15 (StataCorp, 2017) [44] including its update for non-linear decomposition [47], was employed to perform the decomposition analysis. The *normalize* subcommand was used to compute the total contribution of all categories of each categorical variable. All estimates were weighted using the U.S. adult population in 2013, and variances were estimated using the balanced repeated replication method with Fay’s adjustment [34]. Complete case analysis was used for all analyses with an analytical sample of 25,941 participants.

#### Sensitivity analyses

The proportions of missing data for all variables were less than 3% except for annual household income (7.5%). Two sensitivity analyses were conducted to inform the decisions of using complete case analysis including the income variable, despite its relatively high non-response. The first sensitivity analysis was performed by: 1) excluding observations with missing data on annual household income, and 2) excluding the annual household income variable from the set of explanatory factors in the models. When compared to the main analysis using the same observations but including the income variable, the unexplained portion was substantially higher in the sensitivity analysis (joint inequality: 10.1%, referent socio-economic inequality: 52.3%, and referent sexual orientation inequality: 20%) than in the main analysis (joint inequality: − 1.1%, referent socio-economic inequality: 37.9%, and referent sexual orientation inequality: 19.8%), suggesting that annual household income is an important explanatory factor.

The second sensitivity analysis was performed by: 1) including all observations with and without missing data on annual household income, and 2) excluding the annual household income variables from the set of explanatory factors in the models. When compared to the first sensitivity analysis with the same set of variables but excluding the income variable, the results were similar, indicating that excluding observations with missing data on annual household income do not yield biased estimates.

As a result, the final analyses reported in the results section employed complete case analysis and included income in the set of explanatory factors.

## Results

The four intersectional groups differ across all socio-demographic groups (gender, age, race/ethnicity, and marital status). For instance, 60.68% of LGBQ adults with low education were women as compared to 58.74% of LGBQ adults with high education, 51.19% of heterosexual adults with high education, and only 47.43% of heterosexual adults with low education. The largest share of heterosexual adults with low education were aged 45 and above (61.14%) as compared to only 26.85% of LGBQ adults with low education. Additionally, nearly two thirds of LGBQ adults with low education self-identified as Hispanic (64.34%) as compared to only 11.97% of heterosexual adults with high education. More than half of heterosexual adults with high education were married (54.12%) as compared to only 27.99% of LGBQ adults with high education.

The prevalence of current cigarette smoking was unequally distributed between the four intersectional positions (Table 1). High-educated heterosexual adults had the lowest prevalence of cigarette smoking (17.4%), while low-educated heterosexual adults had the highest prevalence of current cigarette smoking (29.7%). High-educated LGBQ adults had higher prevalence of cigarette smoking (27.4%) as compared to low-educated LGBQ adults (23.4%).

**Table 1:** Descriptive statistics of all variables in the total sample and by intersectional positions of sexual orientation and education

	Total	LGBQ adults		Heterosexual adults		*P* value (χ^2^)
		*Low-educated*	*High-educated*	*Low-educated*	*High-educated*	
	Weighted %	Weighted %	Weighted %	Weighted %	Weighted %	
	(unweighted N)	(unweighted N)	(unweighted N)	(unweighted N)	(unweighted N)	
	100 (25,941)	0.79 (301)	4.12 (1,505)	9.72 (3,007)	85.37 (21,128)	
*Socio-demographic factors*						
Gender						< 0.001
Women	51.20 (13,145)	60.68 (204)	58.74 (971)	47.43 (1,355)	51.19(10,479)	
Men	48.80 (13,029)	39.32 (94)	41.26 (533)	52.57 (1,647)	48.81 (10,637)	
Age						< 0.001
18–24	12.58 (7,409)	21.94 (146)	24.26 (625)	11.28 (873)	12.19 (5,721)	
25–44	35.34 (9,401)	51.21 102)	44.98 (595)	27.58 (865)	35.63 (7,751)	
45+	52.08 (9,382)	26.85(53)	30.76(285)	61.14(1,269)	52.18 (7,653)	
Race/ethnicity						< 0.001
Non-Hispanic White	65.85 (15,527)	20.72 (97)	63.51 (855)	43.55 (1,326)	69.15 (13,137)	
Hispanic	15.30 (4,610)	64.34 (132)	19.08 (310)	36.63 (869)	11.97 (3,207)	
Non-Hispanic non-white	18.85 (5,658)	14.95 (66)	18.85 (322)	19.83 (722)	18.88 (4,509)	
Marital status						< 0.001
Married	51.90 (10,089)	38.37 (75)	27.99 (308)	43.29 (982)	54.12 (8,612)	
Separated/ divorced/widow	21.28 (4,962)	20.73 (49)	17.20 (212)	30.88 (759)	20.36 (3,883)	
Never married	26.82 (11,063)	40.91 (172)	54.81 (980)	25.83 (1,248)	25.52 (8,583)	
*Material conditions*						
Annual household income						< 0.001
< $10,000	12.55 (4,822)	49.24 (158)	17.81 (368)	32.55 (1,170)	9.43 (3,047)	
$10,000–$24,999	19.84 (5,891)	30.83 (81)	23.62 (387)	38.87 (934)	17.28 (4,425)	
$25,000–$49,999	22.80 (5,933)	13.95 (37)	22.45 (326)	17.47 (530)	23.58 (4,994)	
$50,000–$99,999	26.11 (5,734)	4.21 (15)	22.41 (276)	8.72 (266)	28.55 (5,129)	
100,000 or more	18.70 (3,815)	1.76 (10)	13.71 (148)	2.40 (107)	21.16 (3,533)	
Employment status						< 0.001
Full-time	49.51 (12,238)	33.93 (81)	50.52 (666)	32.88 (886)	51.64 (10,496)	
Part-time	16.35 (5,134)	23.06 (65)	22.07 (375)	12.51 (488)	16.52 (4,173)	
Unpaid	34.14 (8,731)	43.01 (150)	27.41 (459)	54.61 (1,611)	31.84 (6,406)	
Receiving assistance						< 0.001
Yes	17.75 (5,798)	36.73 (115)	23.82 (432)	32.66 (1,066)	15.53 (4,134)	
No	82.25 (20,357)	63.27 (186)	76.18 (1,071)	67.34 (1,935)	84.47 (16,969)	
Housing						< 0.001
Own	56.19 (10,961)	21.04 (55)	35.71 (385)	40.51 (924)	59.41 (9,498)	
Rent	34.16 (11,139)	65.26 (175)	48.46 (815)	48.84 (1,549)	31.30 (8,481)	
Something else	9.65 (4,015)	13.71 (69)	15.83 (302)	10.65 (518)	9.28 (3,098)	
Health insurance						< 0.001
Private health insurance	66.42 (15,493)	29.72 (93)	60.53 (815)	37.66 (1,063)	70.69 (13,418)	
Medicare or Medicaid	21.15 (6,212)	27.46 (101)	20.42 (372)	38.94 (1,141)	18.85 (4,521)	
No health insurance	12.42 (4,405)	42.82 (104)	19.04 (315)	38.94 (787)	10.46 (3,138)	
*Marketing-related variables*						
Exposure to contextual-level targeting advertisement						0.1655
Yes	37.37 (10,498)	38.68 (137)	42.24 (666)	37.54 (1,239)	37.25 (8,376)	
No	62.63 (15,651)	61.32 (164)	57.76 (839)	62.46 (1,760)	62.75 (12,718)	
Exposure to targeted tobacco marketing						< 0.001
Yes	10.33 (3,514)	6.41 (29)	14.45 (255)	9.07 (356)	10.4 (2,863)	
No	89.67 (22,647)	93.59 (272)	85.55 (1,247)	90.93 (2,645)	89.6 (18,242)	
Exposure to media advertisement						0.0363
Yes	45.93 (12,379)	56.48 (174)	50.12 (759)	47.03 (1,484)	45.59 (9,862)	
No	54.07 (13,764)	43.52 (127)	49.88 (744)	52.97 (1,511)	54.41 (11,231)	
*Intra-personal factors*						
Perceived quality of life						< 0.001
Good	91.16 (23,219)	73.36 (223)	86.13 (1,270)	79.51 (2,355)	92.94 (19,168)	
Fair	7.87 (2,584)	24.41 (66)	11.68 (197)	18.26 (576)	6.32 (1,705)	
Poor	0.97 (372)	2.23 (9)	2.19 (37)	2.23 (72)	0.74 (244)	
Satisfaction with social relations						< 0.001
Very satisfied	67.56 (16,507)	60.17 (163)	55.43 (792)	61.56 (1,720)	68.86 (13,678)	
Moderately satisfied	30.17 (8,877)	35.07 (117)	40.15 (638)	35.16 (1,143)	29.13 (6,896)	
Not at all satisfied	2.27 (778)	4.76 (17)	4.418 (75)	3.29 (137)	2.00 (537)	
Smoking Status						< 0.001
Non-smokers	81.01 (17,147)	76.55 (179)	72.44 (900)	70.25 (1,582)	82.60 (14,310)	
Smokers	18.99 (9,048)	23.45 (122)	27.56 (605)	29.75 (1,425)	17.40 (6,818)	

The subsequent analyses set sought to examine to what degree these inequalities in smoking, specifically the joint and referent socioeconomic and sexual orientation inequalities, were attributable to corresponding inequalities in social processes’ indicators.

### Intersectional inequalities in indicators of social processes

The first set of preliminary analyses (Table 1) illustrate to what degree intersectional social positions are reflected in unequal social processes that may underpin inequalities in smoking, as indicated by material conditions, exposure to marketing, and psychosocial factors, while taking socio-demographic factors into account.

Overall, results in Table 1 indicate that the doubly disadvantaged group of low-educated LGBQ adults exhibited the worse life conditions as compared to the other three intersectional groups, including the highest proportions of low annual household income (49.2%), receiving assistance (36.7%), rented housing (65.3%), lack of health insurance (42.8%), and fair and poor perceived quality of life (respectively 24.4 and 2.2%). Conversely, the doubly advantaged group of high-educated heterosexual adults were better off as compared to the three intersectional groups in terms of high annual household income (21.2%), access to owned housing (59.4%), and access to private insurance (70.7%,).

Interestingly, the singly disadvantaged group of high-educated LGBQ adults had higher proportion of low income (17.8%) and lack of health insurance (19.0%) as compared to high-educated heterosexual adults (9.4%; and 10.5% respectively). High-educated LGBQ adults were also the most exposed group to contextual-level targeting tobacco advertising (42.2%, p, 0.16) and targeted tobacco marketing (15.4%), while low-educated LGBQ adults were the most exposed group to tobacco media advertising (56.5%, p, 0.036).

### Indicators of social processes as predictors of smoking

The second set of preliminary analyses examined to which degree the cited social processes relate to the outcome, current cigarette smoking. Results from three separate multiple logistic regression analyses are reported in Table 2. All three regression analyses were identical expect for the inequality indicator that was included among the set of covariates (joint inequality; referent socio-economic inequality; or referent sexual orientation inequality).

**Table 2:** Summary of adjusted logistic regression

	Joint inequality	Referent socio-economic inequality	Referent sexual orientation inequality
	OR (95% CI)	OR (95% CI)	OR (95% CI)
*Socio-demographic factors*			
*Gender*			
Women	0.67 (0.62, 0.72)	0.65 (0.60, 0.70)	0.68 (0.63, 0.73)
Men	1	1	1
*Age*			
18–24	0.96 (0.83, 1.12)	0.98 (0.85, 1.13)	0.98 (0.84, 1.13)
25–44	1.53 (1.37, 1.71)	1.58 (1.42, 1.74)	1.54 (1.38, 1.71)
45+	1	1	1
*Race/ethnicity*			
Non-Hispanic White	1	1	1
Hispanic	0.44 (0.37, 0.52)	0.38 (0.33, 0.43)	0.45 (0.38, 0.53)
Non-Hispanic non-white	0.64 (0.58, 0.71)	0.67 (0.60, 0.74)	0.64 (0.58, 0.71)
*Marital status*			
Married	1	1	1
Separated/ divorced/widow	1.60 (1.41, 1.83)	1.55 (1.37, 1.76)	1.64 (1.44, 1.87)
Never married	1.44 (1.27, 1.64)	1.41 (1.24, 1.60)	1.47 (1.30, 1.66)
*Material conditions*			
*Income*			
Quintile 1 (lowest income)	2.80 (2.33, 3.37)	2.69 (2.25, 3.21)	2.79 (2.32, 3.36)
Quintile 2	2.52 (2.15, 2.96)	2.33 (2.00, 2.72)	2.44 (2.09, 2.84)
Quintile 3	2.02 (1.72, 2.37)	2.06 (1.76, 2.39)	1.95 (1.68, 2.28)
Quintile 4	1.67 (1.44, 1.92)	1.65 (1.43, 1.89)	1.67 (1.46, 1.91)
Quintile 5 (highest income)	1	1	1
*Employment status*			
Full-time	1	1	1
Part-time	0.69 (0.60, 0.78)	0.72 (0.63, 0.81)	0.68 (0.60, 0.78)
Unpaid	0.75 (0.68, 0.82)	0.74 (0.68, 0.82)	0.76 (0.69, 0.84)
*Receiving assistance*			
Yes	1.23 (1.09, 1.38)	1.27 (1.13, 1.42)	1.24 (1.10, 1.39)
No	1	1	1
*Housing*			
Own	1	1	1
Rent	1.64 (1.48, 1.81)	1.65 (1.50, 1.81)	1.65 (1.50, 1.81)
Something else	1.49 (1.29, 1.71)	1.48 (1.30, 1.67)	1.48 (1.30, 1.67)
*Health insurance*			
Private health insurance	1	1	1
Medicare or Medicaid	1.55 (1.36, 1.76)	1.56 (1.37, 1.77)	1.56 (1.37, 1.77)
No health insurance	2.12 (1.86, 2.43)	2.05 (1.80, 2.34)	2.05 (1.80, 2.34)
*Marketing-related variables*			
*Exposure to contextual-level targeting advertisement*			
Yes	0.98 (0.88, 1.09)	1.02 (0.92, 1.13)	0.99 (0.89, 1.09)
No	1	1	1
*Exposure to targeted tobacco marketing*			
Yes	3.73 (3.34, 4.17)	3.67 (3.29, 4.10)	3.79 (3.41, 4.21)
No	1	1	1
*Exposure to media advertisement*			
Yes	0.72 (0.65, 0.78)	0.72 (0.66, 0.79)	0.71 (0.65, 0.78)
No	1	1	1
*Other factors*			
*Perceived quality of life*			
Good	1	1	1
Fair	2.10 (1.77, 2.49)	2.00 (1.73, 2.33)	2.08 (1.76, 2.44)
Poor	3.11 (2.02, 4.79)	2.60 (1.76, 3.84)	3.01 (1.99, 4.550
*Satisfaction with social relations*			
Very satisfied	1	1	1
Moderately satisfied	1.42 (1.28, 1.57)	1.43 (1.30, 1.58)	1.39 (1.26, 1.53)
Not at all satisfied	1.19 (0.88, 1.62)	1.48 (1.13, 1.94)	1.20 (0.90, 1.61)
*Inequality*	1.01 (0.71, 1.44)^a^	0.65 (0.57, 0.74)^b^	0.82 (0.69, 0.96)^c^

Reference groups for inequality indicators: ^a:^ low-educated LGBQ adults, ^b^: low-educated heterosexual adults, and ^c^: high-educated LGBQ adults

Current cigarette smoking was less common among women, non-Hispanic, or non-Hispanic non-White, but more common among separated, divorced, and unmarried individuals. Smoking was also more common among those of material disadvantage including individuals with low income, receiving assistance, living in rented housing or other arrangements, or having Medicare/Medicaid insurance or no insurance. However, those with part-time or unpaid employment reported less smoking compared to their full-time employed counterparts.

When it comes to the relationship between marketing exposure and smoking, the independent associations were disparate depending on the type of marketing. Adults exposed to individual targeting marketing had nearly 4 times higher odds of cigarette smoking as compared to non-exposed adults (odds ratio (OR) ranged from 3.7 (95% Confidence Interval (CI): 3.3–4.1) to 3.8 (95% CI: 3.4–4.2)), but smoking was less common among individuals reporting exposure to media advertisement, and no association was found to contextual-level targeting, taking all other factors into account.

Adults reporting fair to poor quality of life had considerably higher odds of current cigarette smoking as compared to adults reporting good of life (OR ranged from 2.0 (95% CI: 1.7–2.3) to 3.1 (95% CI: 2.0–4.8)), as did adults moderately satisfied or not at all satisfied with their social relations (OR ranged from 1.2 (95% CI: 0.9–1.6) to 1.48 (95% CI: 1.1–1.9)).

Taken together, material, marketing and psychosocial processes as well as sociodemographic factors were all relevant for explaining smoking patterns, of which low income, lack of health insurance (material) targeted tobacco marketing (marketing), poor quality of life (psychosocial), being woman and Hispanic race/ethnicity (socio-demographic) were the individual indicators that showed the strongest independent association to smoking.

Adjusting for all covariates, indicators of referent socio-economic inequality and referent sexual orientation inequality were significant, whereas, the joint inequality’s indicator was not significant. These inequalities are (were) analyzed in greater detail in the subsequent decomposition analyses.

### The contribution of social processes to joint, referent socio-economic and referent sexual orientation inequality

**Fig. 1:**
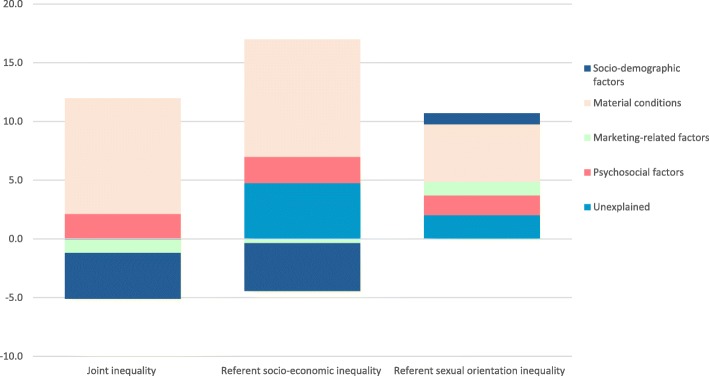
Absolute contributions of explanatory factors to the joint inequality, referent socio-economic inequality, and referent sexual orientation inequality

The final set of analyses sought to estimate to which degree the social processes’ unequal distribution across intersectional social positions on the one hand, and their independent association with smoking on the other, contributed to the intersectional inequalities in smoking. A summary of decomposition analyses is displayed in Table 3, and Fig. 1 shows the absolute contributions of explanatory variables to the inequalities. The models explained sizeable proportions of the inequalities in current cigarette smoking: 101.4% of the joint inequality, 60.1% of the referent socio-economic inequality, and 80.2% of the referent sexual orientation inequality.

**Table 3:** Summary of Blinder-Oaxaca decomposition analyses of joint inequality, referent socioeconomic inequality, and referent sexual orientation inequality in current cigarette smoking

	Joint inequality: low-educated LGBQ adults (group1) vs. high –educated heterosexual adults (group2)			Referent socio-economic inequality: low-educated heterosexual adults (group1) vs. high-educated heterosexual adults (group2)			Referent sexual orientation inequality: high-educated LGBQ adults (group1) vs. high-educated heterosexual adults (group2)		
	Absolute	Relative	P value	Absolute	Relative	P value	Absolute	Relative	P value
Group1	24.21		< 0.001	29.85		< 0.001	27.53		< 0.001
Group2	17.30		< 0.001	17.30		< 0.001	17.30		< 0.001
Difference	6.90		0.015	12.55		< 0.001	10.23		< 0.001
Explained	7.00	101.4	< 0.001	7.80	62.1	< 0.001	8.21	80.2	< 0.001
unexplained	−0.10	−1.4	0.946	4.75	37.9	< 0.001	2.02	19.8	0.025
Contributions									
*Socio-demographic factors*	**−3.90**	**−55.7**		**−4.08**	**−52.3**		**0.93**	**11.3**	
Gender	−0.45	−6.4	0.08	0.31	4.0	0.007	−0.53	−6.4	0.001
Age	0.77	11.1	0.02	−0.74	−9.5	< 0.001	0.59	7.2	0.022
Race/ethnicity	−5.03	−71.9	< 0.001	−4.59	−58.9	< 0.001	−0.80	−9.7	0.001
Marital status	0.81	11.6	0.008	0.94	12.0	< 0.001	1.66	20.3	< 0.001
*Material conditions*	**9.86**	**140.9**		**10.01**	**128.4**		**4.91**	**59.8**	
Annual household income	4.36	62.3	< 0.001	5.03	64.6	< 0.001	1.75	21.3	< 0.001
Employment status	−0.75	−10.8	0.003	−1.04	−13.4	< 0.001	−0.16	−2.0	0.168
Receiving assistance	0.56	7.9	0.012	0.78	10.0	< 0.001	0.28	3.5	0.004
Housing	2.28	32.6	< 0.001	1.77	22.7	< 0.001	1.85	22.5	< 0.001
Health insurance	3.42	48.8	< 0.001	3.47	44.4	< 0.001	1.19	14.5	< 0.001
*Marketing-related variables*	**−1.09**	**−15.5**		**−0.37**	**−4.7**		**1.15**	**14.0**	
Exposure to contextual-level targeting advertisement	0.00	−0.1	0.89	0.00	0.0	0.906	−0.01	−0.1	0.79
Exposure to targeted tobacco marketing	−0.64	−9.2	0.016	−0.29	−3.7	0.189	0.93	11.3	0.004
Exposure to media advertisement	−0.44	−6.2	0.094	−0.08	−1.0	0.501	0.23	2.8	0.076
Psychosocial factors	**2.12**	**30.3**		**2.23**	**28.6**		**1.68**	**20.5**	
Perceived quality of life	1.73	24.8	0.001	1.75	22.4	< 0.001	0.98	11.9	< 0.001
Satisfaction with social relations	0.39	5.5	0.082	0.48	6.2	< 0.001	0.71	8.6	< 0.001

Bold numbers indicate absolute and relative contributions per group of variables (subtotal)

Material conditions made the largest contribution to all the inequalities; the joint inequality (9.8 percentage point (p.p.), 140.9%), referent socio-economic inequality (10.0 p.p., 128.4%), and referent sexual orientation inequality (4.9 p.p., 59.8%). Total and individual contributions exceeding 100% are partly a reflection of the contributions representing point estimates rather than fixed parameters. Moreover, in the presence of counteracting factors, i.e. factors that contributes in the reverse direction (as exemplified by race/ethnicity) and, thus, estimates an inequality that is greater than the one observed in crude analyses, the individual contributions towards the inequality can greatly exceed 100% of the observed total and explained inequality (as exemplified by material conditions for the joint and referent socio-economic inequality).

The high contribution of material conditions to the joint inequality and referent socio-economic reflects the that the gaps in material conditions between the different groups (low-educated LGBQ adults vs. high-educated heterosexual adults and low-educated heterosexual adults vs. high-educated heterosexual adults) are considerable (as seen in Table 1), in combination with several material conditions being strongly related to smoking itself (as reported in Table 2).

This considerable contribution of material conditions to the joint and referent socio-economic inequalities was possible due to a considerable offsetting contribution of certain factors, particularly race/ethnicity, to the joint (− 71.9%) and referent socio-economic (− 58.9%) inequalities. Such seemingly paradoxical contributions were explained by the combination of a high percentage of Hispanic adults among low-educated LGBQ (64.3%; Table 1) and heterosexual (36.6%) adults on the one hand, and a strong negative association between being Hispanic and current cigarette smoking (OR = 0.44–0.45; Table 2) on the other. The offsetting contribution of race/ethnicity and employment status was much less marked for the sexual orientation referent inequality, since the frequency of Hispanics was more similar in high-educated LGBQ (19.1%) and high-educated heterosexual (12.0%) adults.

Among material conditions, annual household income (62.3%), health insurance (48.8%) and housing (32.6%) made the largest contributions to the joint inequality. These contributions were mainly attributed to the 4–5 times higher proportions of low annual household income (49.5%, Table 1) and absence of health insurance (42.8%) among low-educated LGBQ adults, as compared to high-educated heterosexual adults (9.43 and 10.46% respectively). Similarly, the proportion of adults with rented housing among low-educated LGBQ was double (65.3%) that of high-educated heterosexual adults (31.3%). The same material conditions were the most important for the referent socio-economic and sexual orientation inequalities, although their contribution were not as dominant for the referent sexual orientation inequalities mostly due to smaller income inequalities between high-educated heterosexual and LGBQ groups (Table 1).

Psychosocial factors made the second largest contributions to the joint inequality (2.1 p.p., 30.3%), referent socio-economic inequality (2.2 p.p., 28.9%), and referent sexual orientation inequality (1.68 p.p., 20.5%). These contributions came from mainly perceived quality of life, attributed to quality of life being a strong predictor of smoking (Table 2), in combination with fair and poor quality of life being 3–4 times more frequent among low-educated LGBQ (24.4 and 2.2%) and heterosexual adults (18.3 and 2.2%) as compared to high-educated heterosexual adults (6.3 and 0.7%). A similar but less pronounced pattern for quality of life was seen for the referent sexual orientation inequality (11.9% contribution), but for which satisfaction with social relations also contributed moderately (8.6%). This contribution was explained by a higher proportion of not at all satisfied with social relations among high-educated LGBQ (4.4%) as compared to high-educated heterosexual adults (2.0%).

Tobacco marketing-related variables made positive contribution to only referent sexual orientation inequality. This contribution came mainly from exposure to targeted tobacco marketing (11.3%) that made moderately large contributions to this inequality, attributed to exposure to targeted tobacco marketing being considerably more frequent among the high-educated LGBQ than among high-educated heterosexual groups.

In addition to the offsetting contribution of race/ethnicity commented on above, certain of the remaining socio-demographic factors also contributed towards or against the inequality in smoking. Marital status made an important contribution specifically to sexual orientation referent inequality (20.3%), as never being married was more frequent among high-educated LGBQ adults than among high-educated heterosexual groups. Last, gender had offsetting contributions to the joint inequality (− 6.4%, *p* > 0.08) and referent sexual orientation inequality (− 6.4%, *p* < 0.01), and a small significant but significant positive contribution to the referent socio-economic inequalities (4%, *p* < 0.05). In contrast, age made positive contributions to the joint inequality (11.1%, p < 0.05) and the referent sexual orientation inequality (7.2%, p < 0.05), but a small significant negative contribution to the referent socio-economic inequality (− 9.7%, p < 0.05).

## Discussion

The present study examined to what degree intersectional inequalities in smoking by education and sexual orientation could be attributed to inequalities in indicators of multiple social processes. Inequalities in smoking at the intersection of education and sexual orientation are primarily explained by inequalities in material conditions, with a moderate importance of psychosocial factors. More specifically, the joint and the referent socio-economic inequalities were largely attributed to material conditions (annual household income, housing, and health insurance) and to a smaller degree to perceived quality of life. However, annual household income was the main contributor to these inequalities. The referent sexual orientation inequality was additionally explained by marital status (i.e. being single) and exposure to individual-targeting tobacco advertising. These findings are of a significant importance for research, policy, and practice aiming for equity in tobacco control, and by extension also for equity in morbidity and mortality in tobacco-related diseases.

Our study showed that material disadvantage, namely financial disadvantage, is the most universally important social process reinforcing inequalities in cigarette smoking at the intersection of education and sexual orientation. This finding is in accordance with previous studies highlighting the important role of material disadvantage in explaining health inequalities [48, 49]. Moreover, this study expands current knowledge by suggesting that material inequity is not a pathway of relevance merely to socioeconomic inequalities, but is of broader relevance for inequalities in smoking across the entire intersectional space of SES and sexual orientation, including between high-educated heterosexual and LGBQ adults. This points for the need to address financial disadvantage as a way to tackle social inequalities in cigarette smoking more broadly.

The ubiquitous contribution of material disadvantage to intersectional inequalities reflects unequal access to material resources between the doubly advantaged group (high-educated heterosexual adults) as compared to other doubly and singly disadvantaged groups, which may be translated into adoption of risky health behaviors among the disadvantaged groups. This finding suggests that contrary to heterosexual adults, for whom education might confer a certain level of material advantage, LGBQ adults in the U.S. are still facing multiple barriers constraining their access to material resources regardless of their educational level. For example, despite the progress towards adopting policies prohibiting discrimination based on sexual orientation in 33 states in the U.S. [50], sexual orientation discrimination in the workplace is still pervasive and widespread [51], which might limit, among others, LGBQ adults’ access to promotions and work benefits. A comparable relative material disadvantage among LGBQ people, possibly rooted in structural barriers on the labor market and in working life and expressed in health inequalities, have also been noted in northern European contexts [52].

As a contrast to the universal importance of material conditions, one of the interesting findings of our study is the complex role individual-targeted tobacco advertising played in the studied intersectional space. Although strongly related to smoking, it was exclusively important for the referent sexual orientation inequality in smoking, but not for the joint or referent socio-economic inequalities in smoking; which illustrates how one exposure very strongly related to smoking by itself potentially but not necessarily is relevant for the corresponding social inequalities in smoking. Its contribution was rooted in particularly frequent exposure reported by high-educated LGBQ people as compared to the other groups, including low-educated LGBQ people. This may be explained by the different marketing strategies adopted by tobacco companies when targeting different. For example, strategies targeting low SES people [27, 28] may include distributing discount offers at point-of-sale [27], which was not captured by the this study. In contrast, tobacco marketing strategies targeting the LGBQ community may look different [29, 30, 53]; for example, sponsoring LGBQ events and targeting gay bars. Some tobacco brand marketing campaigns have connected tobacco use to LGBQ issues, such as linking freedom to smoke with freedom to marry [29]. More specifically, the higher exposure to individual-targeted tobacco advertising among high-educated LGBQ, instead of LGBQ people more generally, could be associated with stronger participation in the LGBQ community among high SES LGBQ adults [54]. Indeed, affinity [55] and participation in the LGBQ community [56, 57] have been associated with smoking and substance use among LGBQ people. It is also possible that messages that appeal more to well-educated LGBQ people is part of strategic effort by tobacco companies.

Taken together, this specific finding may reflect how different intersectional groups, through specific oppressive social processes, are specifically targeted and exploited for profit, which in turn sheds light on how unexpected and complex population patterns of health-damaging behaviors arise. It, thereby, illustrates the unique knowledge gained from an intersectional approach taking into account both social positions and their expressions in social processes [21].

A further finding warranting a comment is the strong offsetting contribution of race/ethnicity. As we have noted in our previous report, low-educated LGBQ reported less smoking than expected. The decomposition analyses of the present study shows that a large part of this low prevalence of smoking is explained by the high frequency of Hispanic adults, which smoke to a lesser degree than non-Hispanic White adults as also indicated by the national patterns in cigarette smoking among different racial/ethnic groups [5]. Expressed differently, if all ethnic groups smoked equally, the joint inequality would have been considerably larger; 11.9.p.p instead of 6.9 p.p.; and a similarly-sized increase would have been estimated for the SES referent inequality. This suggests that the impact of double socioeconomic and sexual orientation-related disadvantage on smoking might be partially hidden by the outcome-specific protective presence of ethnic groups that nonetheless indeed are socially disadvantaged. This observation illustrates the complexity that comes from an intersectional approach to inequalities in smoking, and is one specific issue that requires further study.

### Study limitations

Our study has several limitations. Despite the importance of psychosocial factors such as perceived discrimination [52] and victimization [14] in explaining socioeconomic inequalities and sexual orientation inequalities in cigarette smoking, these factors were not included in the models. This is mainly due to the absence of variables that might capture these factors in the data set. Similarly, macro-level factors that might explain inequalities in cigarette smoking such as living in areas with smoking-free polices were not included in the models [58]. Decomposition analysis is considered an illustrative method that allows identifying factors contributing to health inequalities. However, this method does not suggest causal inference, which is a particular limitation when using a cross-sectional design, as the present study.

## Conclusions

This study shows that material disadvantage plays a dominant role in explaining inequalities in cigarette smoking affecting not only the doubly disadvantaged group of low-educated LGBQ but also other socioeconomic and sexual orientation groups of single disadvantage. This finding suggests that reducing smoking inequalities in cigarette smoking among U.S. adults more broadly requires addressing the underlying inequalities in material disadvantage among marginalized groups.

## Data Availability

The dataset analyzed in this study is available from: https://www.icpsr.umich.edu/icpsrweb/NAHDAP/studies/36498/datadocumentation.

## Abbreviation

LBGQ: Lesbian, bisexual, gay, and queer/questioning
OR: Odds ratio
p.p.: percentage point
PATH: Population Assessment of Tobacco and Health
SES: Socio-economic status
